# CoQ10 and Vitamin A Supplementation Support Voice Rehabilitation. A Double-Blind, Randomized, Controlled, Three-Period Cross-Over Pilot Study

**DOI:** 10.3389/fphar.2019.00939

**Published:** 2019-09-10

**Authors:** Giovanni Ruoppolo, Lucia Longo, Patrizia Pescerelli, Chiara Mango, Maria Nicastri, Flavia Flaccadoro, Patrizia Mancini, Antonio Greco, Marco De Vincentiis

**Affiliations:** ^1^Department of Sense Organs, Sapienza University of Rome, Rome, Italy; ^2^Department of Oral and Maxillofacial Sciences, Sapienza University of Rome, Rome, Italy

**Keywords:** muscle tension dysphonia, CoQ10, vitamin a, voice rehabilitation, dysphonia severity index

## Abstract

**Objectives:** To evaluate the effectiveness of an adjuvant therapy (CoQ10 in its water-soluble form and vitamin A) in supporting voice rehabilitation in a large group of patients with muscle tension dysphonia (MTD).

**Study Design:** Twelve-week, double-blind, randomized, controlled, three-period cross-over pilot study. The primary endpoint was the change in the Dysphonia Severity Index (DSI) over the 12-week study period. Secondary endpoints were the changes in the subcomponents of DSI, including MPT, F0-high, I-low, and jitter. Exploratory endpoints were the changes in the Shimmer and in Voice Handicap Index (VHI).

**Methods:** Patients were randomly assigned in a 1:1 ratio to two counter-balanced arms. Group A (ADJ-PLA) patients were administered QTer 300 mg and Vit A acetate 500.000 Ul/g 1 mg twice daily for a 4-week intervention period, followed by a 4-week period of wash-out, and then were submitted to a last 4-week period of placebo. Patients in Group B (PLB-ADJ) were given the treatment period in reverse order. Both groups received a 45-min voice therapy in a group format once a day for 4 weeks during the first and the second active periods. The therapy was held during the wash-out period.

**Results:** The analysis of main time effect indicated a trend toward recovery of vocal function regardless of group assignment. A significant time by group effect was found on DSI [*F* = 3.4 (2.5, 80.5), *p* = 0.03], F0-high [*F* = 4.5 (2.6, 82.9), *p* = 0.008] and Shimmer [*F* = 3.6 (1.5, 46.9), *p* = 0.048], under CoQ10 and Vit A treatment, with a small effect size. There was no significant time by group effect on the other study measures, namely MPT, I-low, VHI.

**Conclusions:** A trend toward recovery of vocal function was observed in all the patients, likely due to voice rehabilitation. The improvement of DSI was greater under CoQ10 and Vitamin treatment, indicating a more pronounced improvement of vocal quality under adjuvant therapy. The study protocol was reviewed and approved by the Ethics Committee of Policlinico Umberto I Hospital, Rome, Italy Rif. 3069/13.02.2014.

## Introduction

Functional disorders of voice are frequent in the general population and particularly common among professional voice users. In a study on 2019 patients with dysphonia of different etiologies, functional dysphonia was found in 17% of subjects (Mozzanica et al., 2016), whereas in a Belgic treatment-seeking population, the functional voice disorders were diagnosed in 30% of patients. (Martins et al., 2016). Undoubtedly, the heavy voice demand associated to some professions increases the risk for voice disorders. For example, the reported prevalence of functional dysphonia is of 46% in call center operators and ranges from 20% to 80% in teachers. Occupational voice disorders can involve the social and the economic status of patients and must be considered an important health issue (Hazlett et al., 2011; Niebudek-Bogusz and Śliwińska-Kowalska, 2013).

Functional disorders of voice are defined as alterations of voice quality in absence of identifiable lesions, and there is a general agreement among researchers on their etiology, mainly attributable to an improper voicing behavior. Recently, the main cause of vocal dysfunction has been identified in the increased muscle tension or effort: similar voice pathologies, previously labeled as vocal hyperfunction, hyperkinetic dysphonia, tension-fatigue syndrome, muscle misuse, functional, nonorganic dysphonia, have been grouped together in the broader definition of *primary muscle tension dysphonia (MTD)* (Roy et al., 2019). Voice therapy, aimed both to restore a proper vocal behavior and to treat specific alterations in voice-producing mechanisms, is the first-line treatment of functional voice disorders, even if poor evidence supports its effectiveness (Ruotsalainen et al., 2007). Due to the muscular effort that characterizes the MTD, it is reasonable to assume the usefulness of an adjuvant therapy to support muscle fatigue. Several studies support the efficacy of the ubiquinol, particularly in the reduced and active form of coenzyme Q10 (CoQ10), in improving the performance and decreasing the fatigue in subjects undergoing different workloads (Orlando et al., 2018), as well as improving the impaired myocardial bioenergetics in patients with heart failure and preserved left ventricular ejection fraction (Pierce et al., 2018). Ubiquinol is a lipid-soluble molecule composed of a redox-active quinone ring and a hydrophobic tail. The high concentration of this molecule in the mitochondria confirms its role in the mitochondrial respiratory chain where it acts as a mobile electron transporter. In a recent study on cultured cells (Bergamini et al., 2012), the water-soluble formulation of CoQ10 (Qter) showed to be more efficient than the native CoQ10 in increasing mitochondrial ubiquinone levels, leading to a general improvement of bioenergetics parameters, such as oxygen consumption, ATP content, mitochondrial potential, and protein synthesis. To the best of our knowledge, there is no study in literature about the efficacy of CoQ10 alone on vocal pathologies. With regard to vitamin supplementation, retinoic acid (vitamin A) has been widely used for decades by the otologists to treat laryngeal leucoplakia (Issing et al., 1996) and for the maintenance of the normal structure and texture of the respiratory epithelium. Furthermore, vitamin A was found in the vocal fold stellate cells, which are involved in the metabolism of the extracellular matrix of the lamina propria of the vocal fold, whose viscoelastic properties play an essential role in the vocal fold vibration (Sato et al., 2003; Sato et al., 2004; Sato et al., 2010). Therefore, it can be postulated that the combination of CoQ10 and Vitamin A is useful to support the therapy of functional pathologies of voice. This has been already hypothesized in several reports, which are all limited by the observational design and the lack of the control arm. Of these, only one appears in international databases (Sensini et al., 2011).

The aim of our study was, therefore, to evaluate the effectiveness of CoQ10 in its water-soluble form (Qter) and vitamin A in supporting voice therapy in a large group of patients with MTD, in a 12-week, double-blind, randomized, controlled, three-period cross-over pilot study. The finding of potential benefits of adjuvant therapy could help to shorten and make speech therapy more effective, with a consequent reduction in health expenditure and working hours lost by voice professionals.

The primary endpoint was the change in the Dysphonia Severity Index (DSI) (Wuyts et al., 2000) over the 12-week study period. Secondary endpoints were the changes in the subcomponents of DSI, including MPT, F0-high, I-low, and jitter. Exploratory endpoints were the changes in the Shimmer and in Voice Handicap Index (VHI).

## Materials and Methods

### Participants

Fifty patients referred to the Operative Unit of Phoniatrics of the Azienda Policlinico Umberto I Hospital of Rome for MTD dysphonia were asked to participate in the trial in combination with voice rehabilitation. Inclusion criteria were: 18 years or older and 70 years or younger and a definite diagnosis of MTD without secondary mucosal changes (e.g., epithelial thickening such as nodules or polyps). Exclusion criteria were diagnosis of organic, post-surgical or neurological dysphonia; concomitant diseases (endocrine, metabolic, neurologic disorders) that could be associated with dysphonia; laryngopharyngeal reflux; psychiatric disorders or other conditions that could interfere with the study design. Concurrent diseases and psychiatric disorders were verified using a dedicated checklist where detailed questions were asked. The presence of reflux signs or organic lesions was verified by laryngostrobosocopy. In particular, all patients with a Reflux Finding Score ≥ 2 were excluded (Belafsky et al., 2001). The presence of vocal tremor or other signs of neurological damage was excluded by spectrography. All subjects with alterations in the thyroid hormone metabolism, even if treated, were excluded.

The study protocol was approved by the Ethical Committee of Policlinico Umberto I, Rome, Italy, and was conducted in accordance with the International Conference on Harmonization Guidelines for Good Clinical Practice and the declaration of Helsinki. Each patient provided written informed consent before any study-related procedure.

### Study Design

Patients who met the eligibility criteria underwent study assessments and were randomly assigned in a 1:1 ratio to two counter-balanced arms (group A–group B) by computer-generated random numbers. An operator not involved in study measurements performed the randomization procedure. Both patients and operators were blinded to assignments.

**Figure 1:**
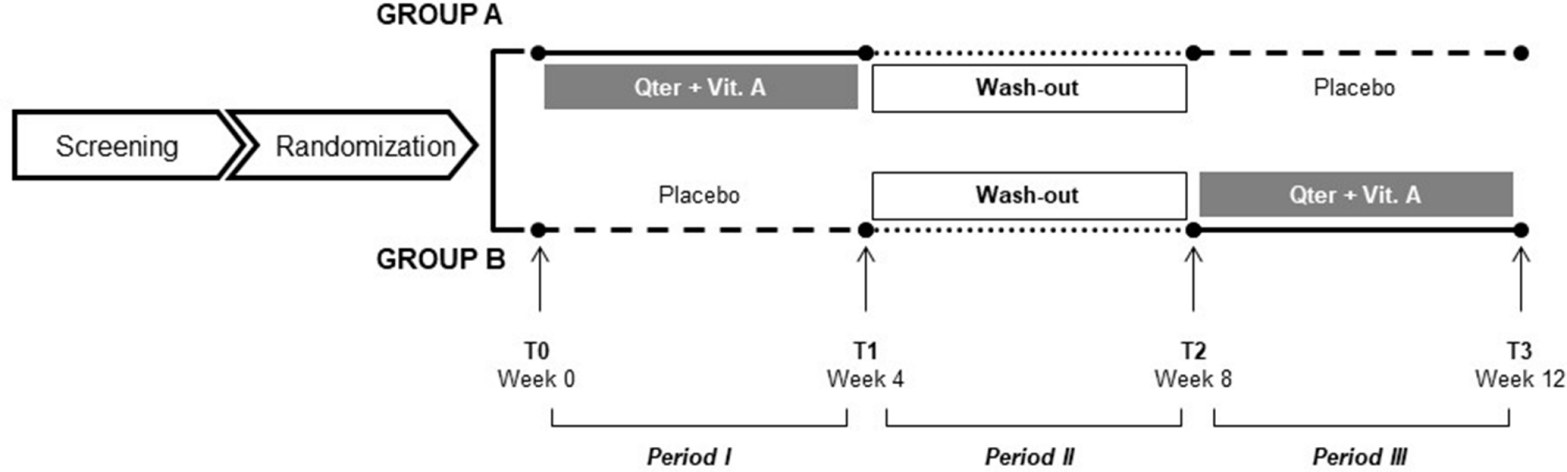
Study design.

Group A (ADJ-PLA) patients were administered QTer 300 mg (equal to CoQ10 30 mg) and Vit A acetate 500,000 Ul/g 1 mg twice daily for a 4-week intervention period, followed by a 4-week period of wash-out, and then were submitted to a last 4-week period of placebo (inert tablets resembling the active medication). Patients in group B (PLB-ADJ) were given the treatment period in reverse order. Both groups received an intensive 45-min voice therapy in a group format (six participants) once a day from Monday to Friday for 4 weeks during the first and the second active periods. The therapy was held during the wash-out period (**Figure 1**).

Patients were encouraged to contact the responsible study person (RG) in case of any adverse event (defined as any untoward medical occurrence regardless of its causal relationship to the study intervention) that could occur during the 12-week study period.

Study assessments were conducted at randomization (T0), after the first 4-week period (T1), at the end of the wash-out period (T2), and finally at the end of the 12-week study period (T3).

### Study Assessments

All patients underwent a comprehensive voice examination as follows:

- *Dysphonia Severity Index* (DSI): DSI is a validated multiparametric indicator for the assessment of the severity of dysphonia and a suitable method for dysphonia quantification (Hakkesteegt et al., 2008; Hakkesteegt et al., 2010; Van Lierde et al., 2010). It is calculated by means of a weighted combination of the highest possible frequency (F0_high_), the lowest intensity (I_low_), Maximum Phonation Time (MPT), and jitter% (an acoustic analysis of relative pitch disturbance), using the following formula: 0.13 MPT + 0.0053 F_high_ - 0.26 I_low_ - 1.18 jitter (%) + 12.4. The DSI for perceptually normal voices equals +5 (or 100%) and for severely dysphonic voices equals -5 (or 0%). The more negative the patient’s index, the worse is the voice quality.
- *Shimmer%*: acoustic analysis of relative amplitude disturbance.
- *MPT*: maximum phonation time is the longest period during which a patient, after taking a deep breath, can sustain phonation of a vowel sound. In dysphonic patients, it is used also to objectively assess the progresses after voice therapy (Maslan et al., 2011). MPT was measured by means of three tests using the vowel/a/, sustained at the subject’s habitual pitch and loudness in a standing position.
- F0_high_: it is the highest frequency of the subject’s voice. The patient was asked to vocalize the vowel “a” with the highest tone possible.
- *I*_low_: it is the lowest intensity of the subject’s voice, measured by means of a phonometer (Bruel & Kjaer type 2232) at 30 cm. The patient was invited to vocalize the vowel “a” as much softly as possible.

For both F0_high_ and *I*_low_ the average of three tests was considered.

*VHI*: developed and validated by Jacobson in 1997 (Jacobson et al., 1997) to assess patients’ perception of the severity of their voice, it consists of three domains, including functional, physical, and emotional aspects of voice disorders and is administered by patients themselves in a five-point Likert-type scale manner for each item (from 0, never to 4, always). We used the Italian validated version (Schindler et al., 2010).

Voice examination was carried out by a senior ENT specialist (RG) experienced in voice diagnostics. He was aware of the timing of the study but blind to treatment allocation. The acoustical analysis was carried out by means of a personal computer, used to operate an MDVP module which acquires, analyzes, and displays voice parameters using Visi-Pitch IV hardware system. The subject was seated in a quiet room (environmental noise <30 dB, monitored through the phonometer Bruel & Kjaer type 2232) and instructed to phonate three times, for 3 s, a sustained “a” to a microphone (Shure cardioid PGA58-XLR) at a comfortable pitch and amplitude. The patient was then requested to repeat three times the phonation at the higher possible frequency.

### Voice Therapy

Voice therapy was provided in a group format by a senior speech language pathologist (SLP) with over 25 years of experience in voice rehabilitation (PP), assisted by two graduated SLP practitioners (MC, FF). All of them conducted group therapy together. Voice rehabilitation consisted of an individually tailored combination of indirect and direct techniques. Indirect techniques were aimed at increasing the awareness of vocal effort, improving vocal hygiene, and eliminating vocal abuse. Direct methods included diaphragmatic breathing, coordination of breathing with phonation and laryngeal relaxation, with attention to reduce the excessive contraction of laryngeal muscles and minimize the inappropriate co-contraction of the muscles of vocal tract while engaged in phonation. The optimal use of resonance and the control of pitch and loudness in reading and in spontaneous conversation was finally pursued. Our hospital provides intensive voice therapy both to maximize the individual’s ability to learn and carryover targets to non-clinical environments and to enable patients to return to work as soon as possible with an adequate voice (Patel et al., 2011; Fu et al., 2015).

### Statistical Analysis

Given the exploratory nature of this pilot trial, no sample size analysis was performed. Data are presented as mean (standard deviation) or median (range) as appropriate.

Well-balancing of two treatment groups after randomization were tested using the Mann–Whitney *U* test or the Fisher’s exact test for continuous and categorical variables, respectively.

Repeated measures analysis of variance (RM-ANOVA) was conducted to explore the effect of Qter and Vit A in addition to voice rehabilitation on vocal function. Each RM-ANOVA model included the raw scores derived from each assessment as dependent variable, the study visits (T0, T1, T2, and T3) as within-subject factor, and treatment groups (VOC-PLC and PLC-VOC) as between-subject factor. The main effect of time by group interaction, together with effects size based on Cohen’s *f*-squared, was provided to explore the efficacy of Qter and Vit A in improving vocal function. Effect sizes of 0.02, 0.15, and 0.35 were termed small, medium, and large, respectively. We considered significant a *p* value < 0.05 in either direction. Statistical analyses were performed with the Statistical Package for Social Sciences, version 16.0 (IBM SPSS, Chicago, IL, USA).

## Results

### Participants

From September 2017 to January 2018, a total of 50 patients were assessed for eligibility. Two patients declined to participate, whereas nine did not meet the inclusion criteria. Thirty-nine patients (27 females, 12 males), with a mean age of 50 (15) years, were randomized into two counterbalanced group as follows: 21 were assigned to group A (VOC-PLB) and 18 to group B (PLB-VOC). The two treatment groups were similar in terms of baseline demographics and clinical characteristics (*p* ≥0.11 for all comparisons). Considering separately for males and females, the starting values of F0-high and MPT were not statistically different (*p* ≥ 0.14) (**Table 1**).

**Table 1:** Baseline characteristics of study sample (n = 39).

	Whole sample (N = 39)	VOC-PLB (n = 21)	PLB-VOC (n = 18)	p
Sex F:M	27:12	15:6	12:6	0.75
Age, years	50.1 (14.6)	46.9 (13.0)	53.7 (15.7)	0.19
DSI	-0.96 (2.41)	-1.24 (2.47)	-0.63 (2.37)	0.30
MPT, s	12.1 (3.6)	11.4 (3.6)	12.9 (3.4)	0.14
MPT F, s		10.9 (3.1)	12.4 (3.2)	0.28
MPT M, s		13.3 (4.9)	13.0 (3.6)	0.97
F_0_-high, Hz	248.4 (60.9)	237.5 (51.7)	261.2 (69.5)	0.38
F_0_- high F, Hz		247.9 (43.4)	291.6 (59.48)	0.14
F0- high M, Hz		183.2 (47.4)	200.5 (37.9)	0.52
I-low, dB	57.4 (5.4)	56.8 (4.4)	58.1 (6.4)	0.59
Jitter, %	1.24 (1.06)	1.37 (1.35)	1.08 (0.55)	0.76
VHI	12.8 (6.8)	10.7 (6.6)	13.2 (6.4)	0.11

F, female; M, male; DSI, Dysphonia Severity Index; MPT, maximum phonation time; F_0_-high, highest phonational frequency; I-low, lowest intensity; VHI, Voice Handicap Index.

All values are mean (SD) unless indicated otherwise.

**Figure 2:**
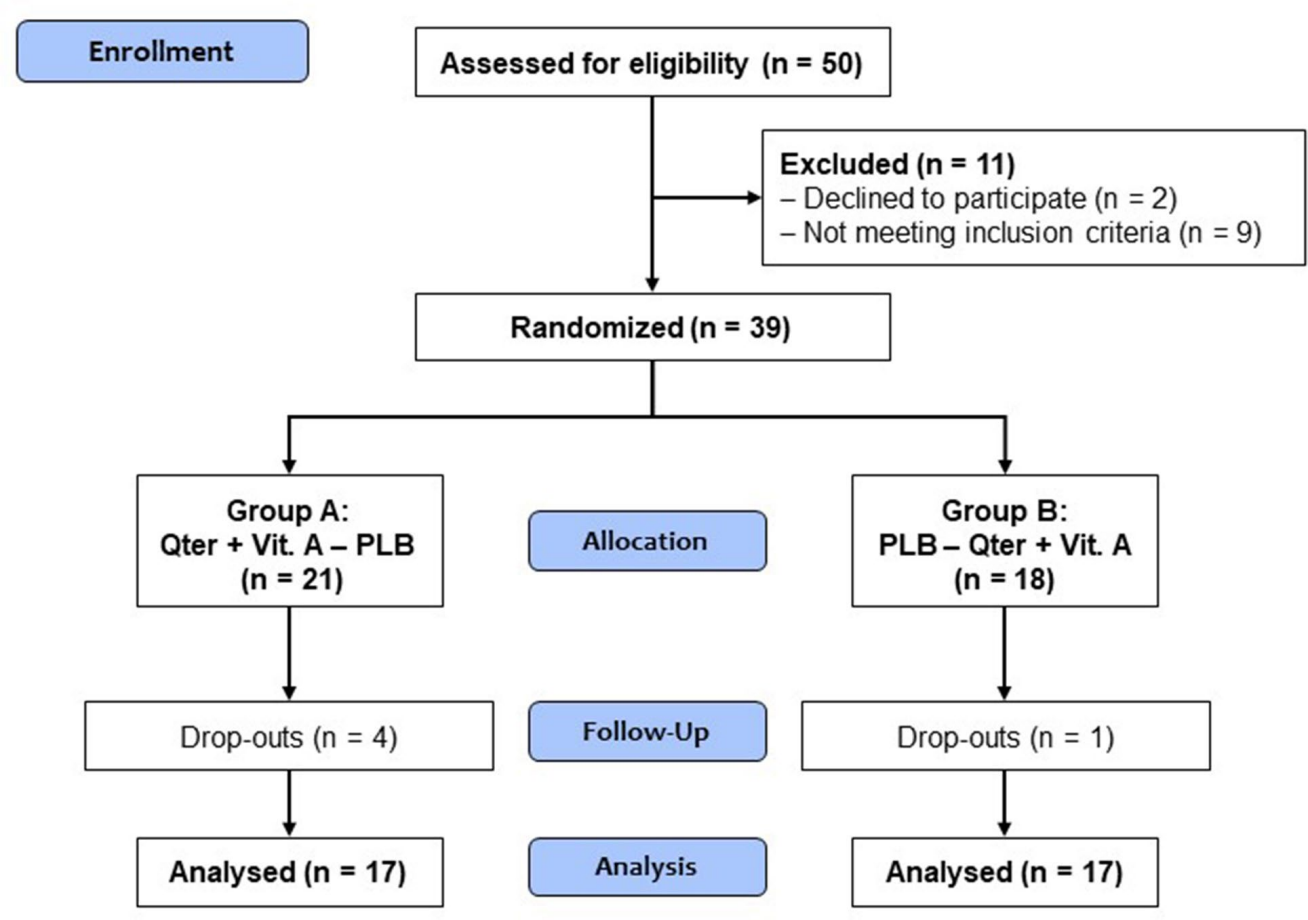
Study flow diagram.

Five patients (four in group A and one in group B) dropped out just after completing the first study period (visits T0 and T1), thus leading to missing data in the following study periods (study visits T2 and T3). Therefore, they were excluded from the case-base analysis according to a listwise deletion procedure. However, sensitivity analyses were done by replacing missing values relative to these five patients according to a “linear trend at point” regression method, i.e., the existing series was regressed on an index variable scaled 1 to n, and missing data were replaced with their predicted values. The study flow diagram is depicted in **Figure 2**.

### Main Findings

Mauchly’s test indicated that the sphericity assumption had been violated for all study measures [χ^2^ (2) ranging from 25.6 to 8.1, *p* <0.05]; therefore, the degrees of freedom were corrected by the Huynh-Feldt method.

The case-base analysis was performed on 34 patients after having excluded the cases with missing data and showed significant main effects of time for all measures, with *F* values ranging from 19.5 to 3.2 (*p* <0.05). The jitter was slightly reduced in both groups in the active period only.

Overall, the analysis of main time effect indicates a trend toward recovery of vocal function regardless of group assignment, likely driven by voice rehabilitation.

**Figure 3:**
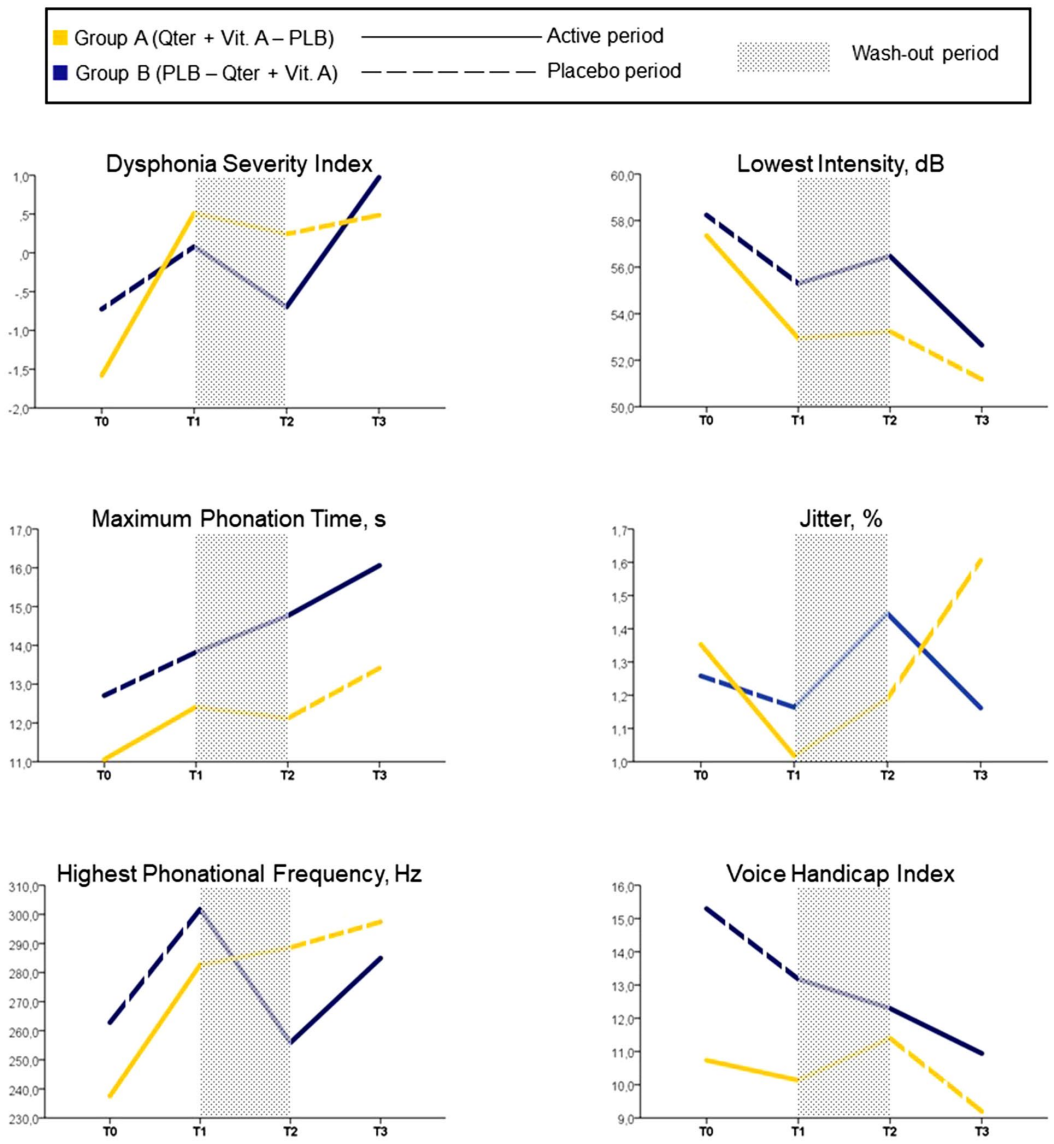
Mean values of study assessments collected over the 12-week follow-up period (case-base analysis, n = 34).

We found a significant time by group effect on DSI [*F* = 3.4 (2.5, 80.5), *p* = 0.03], F0-high [*F* = 4.5 (2.6, 82.9), *p* = 0.008] and Shimmer [*F* = 3.6 (1.5, 46.9), *p* = 0.048], indicating a more pronounced improvement of vocal function under Qter and Vit A treatment, with a small effect size (**Table 2**). There was no significant time by group effect on the other study measures, namely MPT, I-low, VHI (**Figure 3**).

**Table 2:** Summary of the main study findings reporting the effect of time by group interaction (estimated by RM-ANOVAs) in the case-base analysis (n = 35) and sensitivity analysis (n = 39).

		Case-base analysis		Sensitivity analysis
DSI	*F*_[2.5, 80.5]_	3.39	*F*_[2.3, 84.5]_	2.79
	P	**0.03**	p	0.06
	ES	0.08	ES	0.04
MPT, s	*F*_[2.8, 90.4]_	0.71	*F*_[3.0, 95.8]_	0.50
	P	0.54	p	0.66
	ES	0.02	ES	0.01
F_0_-high, Hz	*F*_[2.6, 82.9]_	4.49	*F*_[2.4, 89.5]_	3.21
	P	**0.008**	p	**0.03**
	ES	0.12	ES	0.08
I-low, dB	*F*_[2.5, 81.3]_	0.87	*F*_[2.4, 88.2]_	0.55
	P	0.44	p	0.61
	ES	0.02	ES	0.01
Jitter, %	*F*_[2.4, 75.3]_	1.65	*F*_[2.3, 84.1]_	1.68
	P	0.19	P	0.19
	ES	0.05	ES	0.05
VHI	*F*_[2.3, 69.9]_	1.39	*F*_[2.1, 75.4]_	1.13
	P	0.25	P	0.33
	ES	0.04	ES	0.03
Shimmer,%	*F*_[1.5, 46.9]_	3.58	*F*_[1.6, 57.2]_	4.33
	P	**0.048**	P	**0.026**
	ES	0.07	ES	0.08

DSI, Dsyphonia Severity Index; MPT, maximum phonation time; F0-high, highest phonational frequency; I-low, lowest intensity; VHI, Voice Handicap Index.

Bold values denote statistical significance.

Sensitivity analysis (performed after replacing missing values from five patients who dropped out) showed no relevant difference with respect to the case-base scenario. We confirmed significant main effect of time for all measures (*F* values ranging from 19.2 to 3.4, *p* <0.05) with the exception of jitter [*F*(2.3, 84.1) = 1.31, *p* = 0.21]. Time by group effects were nearly significant on DSI [*F*(2.3, 84.5) = 2.8, *p* = 0.06] and significant on F0-high [*F*(2.4, 89.5) = 3.2, *p* = 0.03] and Shimmer% [*F*(1.6, 57.2) = 4.33, *p* = 0.026] (**Table 2**).

No adverse events were reported.

## Discussion

The primary endpoint of our study was the change in the DSI over the 12-week study period. Although a trend toward recovery of vocal function was observed in all the patients, likely due to voice rehabilitation, greater and significant changes on DSI (*p* = 0.03) were found in the experimental group, showing a substantial effect of Qter and Vit A on vocal improvement of patients undergoing voice therapy. MTD is characterized by the hypercontraction of the laryngeal musculature which reduces the vocal efficiency thus forcing the patient into an increased muscular effort, with establishment of a vicious circle. We can assume that the increased levels of Qter may have improved the bioenergetic functions of the respiratory and phonatory system, allowing a better voice recovery in the experimental groups.

With regard to the secondary outcomes, we found a significant increase of the F0-high (*p* = 0.008). This parameter is not enough, in itself, to show an improvement of vocal quality, but its increase confirms the improved vibration of the vocal folds at the high frequencies. This could be explained by the beneficial effects of Vit A in restoring the normal texture of the laryngeal mucosa. Vocal folds are layered structures, consisting of an inner muscular layer (the thyroarytenoid muscle), a soft tissue layer of the lamina propria, and an outmost epithelium layer. When using the “chest voice” at the lower frequencies, all the structures of the vocal fold are involved in the vibration, whereas the “falsetto” voice is mainly due to the uncoupled vibration of the epithelium layer only. So, the high-frequency vibration requires the perfect efficiency of the biomechanical properties of the epithelium and of the extracellular matrix, to which Vit A contributes. The highest frequency is among the chosen parameters of DSI since every alteration of the vocal mucosa hampers the higher vibratory rates, which is reflected by a decreased F0 (15). The jitter% values showed also a reduction in the active periods for both groups, although failed to reach the statistical significance. This may be due to the small sample size. It should be noted that the Shimmer% values, one of our exploratory endpoints, showed a significant reduction in the active groups (*p* = 0.048). The improvement of both MPT and VHI did not reach the statistical significance. In our clinical experience however, the increased awareness of the vocal effort after voice therapy usually results in the reluctance of patients to maintain a sustained phonation for a long time and in judging more severely the consequences of their dysphonia. This could explain such results, an exploration of data from larger series of patients who underwent voice rehabilitation in the future will shed more light on this observation. Furthermore, if the short time elapsed from the baseline (3 months) has been enough to allow a first improvement of voice quality following rehabilitation, we can assume that patients enduring over time in the correct vocal behavior will achieve even better phoniatric results. The intensive voice treatment can justify the generalized improvement in vocal function but does not affect results, due to the methodology of the study, randomized and conducted in a three-period cross-over.

Unlike previous reports, this is the first double-blind, randomized, controlled, three-period cross-over study about the efficacy of the adjuvant therapy on voice recovery. Most likely, a larger sample size would have allowed to achieve a more profound statistical significance, particularly on data from sensitivity analysis. We encountered great difficulties both in recruiting patients willing to undergo a prolonged voice therapy during working hours and in selecting patients who met the inclusion criteria, particularly without laryngo-pharyngeal reflux signs or epithelial thickening of the vocal folds. Most patients did not agree to undergo plasma analysis, which would have resulted in stronger data. Another limitation of our study could be that we have not analyzed data from laryngoscopy. This choice was motivated by the difficult interpretation of their clinical value—signs of vocal hyperfunction were found also in asymptomatic subjects (Morrison et al., 1983; Belafsky et al., 2002)—and by the greater complexity of statistical processing due to their inclusion, without the addition of objective information. For the same reason we did not use the most widely accepted perceptual judgment protocol, namely the GIRBAS, which is affected by several sources of variability, such as the professional experience of the examiner and the type of examined sample (Dejonckere et al., 1993). On the contrary, the aerodynamic measures are objective, and they would have been useful in evaluating the increased phonatory flow in patients suffering from MTD. However, they are invasive, not easy to perform, and may cause discomfort to the patients. So, the only aerodynamic measure we used was the MPT, which is routinely employed in the assessment of voice functionality and is included in the DSI. Ultimately, we have chosen the DSI as a reference value to assess the voice to facilitate statistical data processing, because it allows the use a single parameter to define the quality of a multidimensional function, as the voice is. DSI indeed includes voice range profiles, aerodynamic, and acoustic measurement.

In conclusion from our double-blind randomized, three-period cross-over pilot study, a combination of CoQ10 and vitamin A was confirmed to be a useful adjuvant therapy in voice rehabilitation.

## Ethics Statement

This study was carried out in accordance with the recommendations of “name of guidelines, name of committee” with written informed consent from all subjects. All subjects gave written informed consent in accordance with the Declaration of Helsinki. The protocol was approved by the “name of committee.”

## Author Contributions

GR, LL, PM, and MN contributed to the design of the study, interpretation of the data, preparation, and approval of the manuscript. PP, CM, FF, and MN contributed to the data collection, analysis, and interpretation of the data. MD and AG contributed to the review and approval of the manuscript.

## Funding

This work is supported by Scharper Spa, Milan, Italy.

## Conflict of Interest Statement

The authors declare that this study received funding from Scharper Spa, Milan, Italy. The funder participated only in the design of the study and in the provision of the placebo. The funder was not involved in data collection or analysis.
